# Effect of cognitive behavior therapy (CBT) on lowering of blood glucose levels in gestational diabetes mellitus (GDM) patients: study protocol for a prospective, open-label, randomized controlled trial

**DOI:** 10.1186/s13063-022-07060-8

**Published:** 2023-01-14

**Authors:** Ying Pan, Hong-ying Liu, Shao Zhong

**Affiliations:** 1 First People’s Hospital of Kunshan Affiliated With Jiangsu University, Kunshan, Jiangsu Province China; 2 Hangzhou Kang Sheng Health Consulting CO., LTD, Hangzhou, China

**Keywords:** Gestational diabetes mellitus, Mobile-based cognitive behavior therapy, Hyperglycemia, Infant outcomes, Maternal outcomes

## Abstract

**Background:**

Gestational diabetes mellitus (GDM) is a common perinatal condition. Convincing evidence has shown that hyperglycemia and other chronic comorbidities of diabetes during the prenatal period increase maternal and fetal risk. Several guidelines have identified lifestyle management as the first-line therapy in GDM patients. To improve the efficacy of lifestyle intervention, cognitive behavior therapy (CBT) is proposed as a solution to improve clinical outcomes. The objective of this trial is to determine the efficacy in treating hyperglycemia of mobile-based CBT interventions in GDM patients, compared with conventional face-to-face interventions.

**Methods:**

This trial is designed as a prospective randomized controlled trial, which enrolled the patients diagnosed with GDM in First People’s Hospital of Kunshan affiliated with Jiangsu University from September 2021 to March 2023 with a 3-month follow-up. The specific randomization method was established and implemented through the central randomization system of EDC clinical trials. The percentage of all blood glucose levels collected within the normal range between the two groups at baseline, during the intervention period, and postpartum infant and maternal outcomes will be measured. Summary statistics for continuous variables will include the number of subjects, mean, median, SD, or the standard error, minimum, and maximum. The chi-square test, *t* test, and paired-sample *t* test were used for statistical analysis of differences between groups.

**Discussion:**

This trial investigates the effects of mobile-based CBT intervention on blood glucose levels in GDM patients.

**Trial registration:**

Chinese Clinical Trial Registry (ChiCTR2100048527) [registered: 2021/07/09].

## Strengths

- We propose an approach in which researchers and participants are involved throughout the entire process, from data collection to the action plan. The algorithms of the system are developed by collecting real patient data, establishing disease databases and disease models. Accordingly, this system will accommodate the Chinese population more appropriately.
- Traditional CBT intervention is delivered face-to-face, which has the limitation of time-consuming and location-specific. Our mobile-based intervention system is developed independently by a professional team based on the theories of computerized CBT. It is characterized by a self-service intervention method. This allows our CBT intervention to overcome these limitations and makes it more accessible to the patients.
- It is expected to benefit the patients when considering the cycle of disease in disease management. From the perspective of a patient’s journey, this intervention realizes systemic management of disease with a seamless connection between in-hospital care and home-based care. As a result, this can bring more value to the patients.

## Limitations

- This study excludes pregnant women whose past medical history include diabetes and only investigates women developing diabetes after pregnancy. Future studies could involve a larger patient group.
- Due to the nature of the research, it is impossible to blind participants and staff (excluding statisticians) in psychotherapy studies. Consequently, therapy expectancy effects may be a source of bias.

## Administrative information

**Table:** 

**Title**	Effect of Cognitive Behavior Therapy (CBT) on Lowering of Blood Glucose Levels in Gestational Diabetes Mellitus (GDM) Patients: Study Protocol for a Prospective, Randomized Controlled Trial
**Trial registration**	Chinese Clinical Trial Registry (ChiCTR2100048527) [registered: 2021/07/09]
**Protocol version**	2021-September-20: Original; version 1.1
**Funding**	Zhiyun Health “2021 Digital Technology Innovation Foundation”) will only consider applications that fall within the objective and policies of the funding program. It particularly aims to pump prime research, and to support researchers in the early phases of their career. “2021 Digital Technology Innovation Foundation” will fund research into GDM diseases primarily within the endocrine department of the First People’s Hospital of Kunshan affiliated with Jiangsu University typically makes awards of between 10–20 thousands RMB Funding is received from Hangzhou Kang Sheng Health Consulting CO,. Ltd
**Author details**	Ying Pan: Hong-ying Liu: hongyingliu@91jkys.com, Shao Zhong: drzhong@163.com; YP and SZ are affiliated with the First People’s Hospital of Kunshan affiliated with Jiangsu University, Kunshan, Jiangsu Province, China. HL is affiliated with Hangzhou Kang Sheng Health Consulting CO., LTD
**Name and contact information for the trial sponsor**	Jia Tang, jiatang@91jkys.com; Hangzhou Kang Sheng Health Consulting CO., LTD
**Role of sponsor**	Hangzhou Kang Sheng Health Consulting CO., LTD provides the technology to design the MiniApp “GDM CBT” used in interventions

## Introduction

Gestational diabetes mellitus (GDM) is a common perinatal condition accompanied by glucose intolerance throughout pregnancy. Convincing evidence has shown that hyperglycemia and other chronic comorbidities of diabetes during the prenatal period increase maternal and fetal risk, including spontaneous abortion, fetal abnormalities, and pre-eclampsia [1, 2] Several guidelines have identified lifestyle management as the first-line therapy in GDM patients [3–5]. Due to the fact that most oral anti-diabetic medications cross the placenta or lack safety data in treating GDM, there is a considerable demand for a non-pharmacological treatment of GDM patients. For most patients, lifestyle management is sufficient to treat GDM [4, 5]. Insulin should be considered to achieve glycemic goals if necessary [3, 6]. Currently, nutrition and physical activity counseling are primary tools for lifestyle intervention. However, the gap between hospital and home has restricted continuous and thorough treatment and follow-up. Considering how to provide lifestyle interventions to promote better clinical outcomes, we sought to determine an effective method to provide GDM patients with efficient and structured lifestyle interventions [5].

Cognitive behavior therapy (CBT) is a goal-directed and systematic psychological therapy created by A.T. Beck in the 1960s to manage out-of-control emotions, behavior, and cognition [7]. CBT focuses on increasing self-efficacy through cognitive and behavioral changes, which benefits patients in terms of clinical results [8]. In GDM patients, glucose management involves everyday decision-making, including food choices, appropriate physical activities, monitoring blood sugar, and stress management [9]. Previous study has suggested that participants who become aware of their unhealthy behavior will intrinsically be driven to change. In these patients, clinical outcomes are improved with practical tools for long-term proactive behavioral change [10]. With regard to what we utilize as our tools, we suppose lifestyle management delivered with CBT will benefit GDM patients from the positive impact on compliance and long-term performance, and we consider that mobile-based CBT can potentially improve clinical outcomes in GDM patients.

Major components of lifestyle modification in GDM patients include nutrition therapy and weight management [3, 11]. These factors depend on patients’ self-efficacy, which theoretically can be improved by computerized CBT. In consequence, we suppose that CBT-based lifestyle intervention is effective in reducing glucose levels in GDM patients.

### Evidence before this study

Improved self-efficacy is a key component of lifestyle adjustment, which has been correlated with improved clinical results in GDM patients. Traditionally, patients are seen in an outpatient setting where healthcare experts can deliver lifestyle interventions to them face to face. However, the limitation of time-consuming and location-specific has been an obstacle for GDM patients to receive a consistent intervention. To encourage healthy lifestyles, novel intervention methods have been developed, including mobile-based interventions. We searched PubMed for researches that had investigated the use of CBT in diabetic patients from November 2016 to November 2021. Search terms included diabetes, gestational, pregnancy, mobile-based, and cognitive behavioral therapy. MeSH (Medical Subject Headings) was used to include synonyms. Based on our review, several studies have shown that CBT is effective in improving subjective data such as depression and social and occupational functioning in diabetic patients, but none was found to investigate objective findings in GDM populations. We identified two randomized controlled studies that have investigated the efficacy of CBT in T2DM patients. The results prove that CBT interventions significantly reduce diabetes-related distress (DRD) and hemoglobin A1c (HbA1c) compared with the control group (*n* = 56, *n* = 222) [12, 13]. Another randomized controlled trial has investigated the efficacy of lifestyle interventions in reducing the risk of developing diabetes and cardiovascular disease. Evidence has shown that compared with health brochures, lifestyle intervention such as motivational interview was not more effective in reducing the risk in an at-risk population (*n* = 622) [14].

### Added-value of this study

Most of the previous studies target T2DM patients, but there is limited information on CBT’s effect on GDM populations, who are recommended to be treated with lifestyle intervention as first-line therapy [3–5]. In addition, our study is designed to evaluate CBT’s efficacy in infant and maternal outcomes. Researchers have attributed GDM to an increased risk of fetal adverse events, including neonatal infection [15]. This study will further evaluate the efficacy in parameters measuring these outcomes, such as the case number of low-birth-weight neonates and comorbidity of GDM in participants.

### Objectives

Aim 1: To determine the efficacy in treating hyperglycemia of mobile-based CBT interventions in GDM patients, compared with conventional face-to-face interventions.

- Hypothesis 1a (primary study hypothesis): a significant difference in the percentage of blood glucose levels within a normal range is expected between the two groups. The normal range is defined as fasting blood glucose.

Aim 2: To determine the efficacy of mobile-based CBT interventions in infants and maternal outcomes, compared with conventional face-to-face interventions.

- Hypothesis 2a: a significant difference in the variables evaluating infants and participants outcomes, including low-birth-weight neonates, neonatal macrosomia, events of hypoglycemic events in infants, and cesarean delivery, is expected between two groups.

### Trial design

This study is a single-center, randomized, controlled, open-label parallel-group superiority trial. Patients will be randomly allocated at a 1:1 ratio to evaluate the efficacy of CBT to lower blood glucose in GDM patients. The study protocol follows the Standard Protocol Items: Recommendations for Interventional Trials (SPIRIT) [16].

## Methods: participants, interventions, and outcomes

### Study setting

The study will be performed at the First People’s Hospital of Kunshan affiliated with Jiangsu University, Kunshan, Jiangsu Province, China, from September 2021 to March 2023.

### Eligibility criteria

#### Inclusion criteria

Patients’ inclusion criteria will be as follows: (1) age from 18 to 45 years old; (2) pregnant women who completed oral glucose tolerance tests (OGTT) at 24–28 gestational weeks; (3) meet diagnostic criteria of GDM and has not received pharmacological therapy; (4) no difficulty in using smartphones, basic Chinese reading and writing skills, basic calculation skills; (5) agree to sign the informed consent form.

#### Exclusion criteria

Patients’ exclusion criteria will be as follows: (1) prior diagnosis of diabetes before pregnancy; (2) prior diagnosis of GDM in previous pregnancy; (3) diagnosed with severe mental disorders or has difficulty understanding; (4) other conditions that could prevent the patients from participating in the research. This trial does not exclude any other concurrent therapies or interventions. The number and doses of insulin prescriptions will be recorded.

Researchers will perform an initial eligibility screening and collect written informed consent from enrolled participants. Written informed consent will be collected prior to the baseline assessment. If the data collection is not included in the initial informed consent process for the main clinical trial, each participant in the ancillary study must sign a consent form.

### Interventions

To compare the effects of CBT in GDM patients, a control intervention—a conventional lifestyle intervention—will be provided. Patient education will be provided to the control group via oral communications from endocrinologists. The control group will also receive patient education, which will include disease-related knowledge, lifestyle modification, complication prevention, and other topics, but only during each follow-up.

**Fig. 1:**
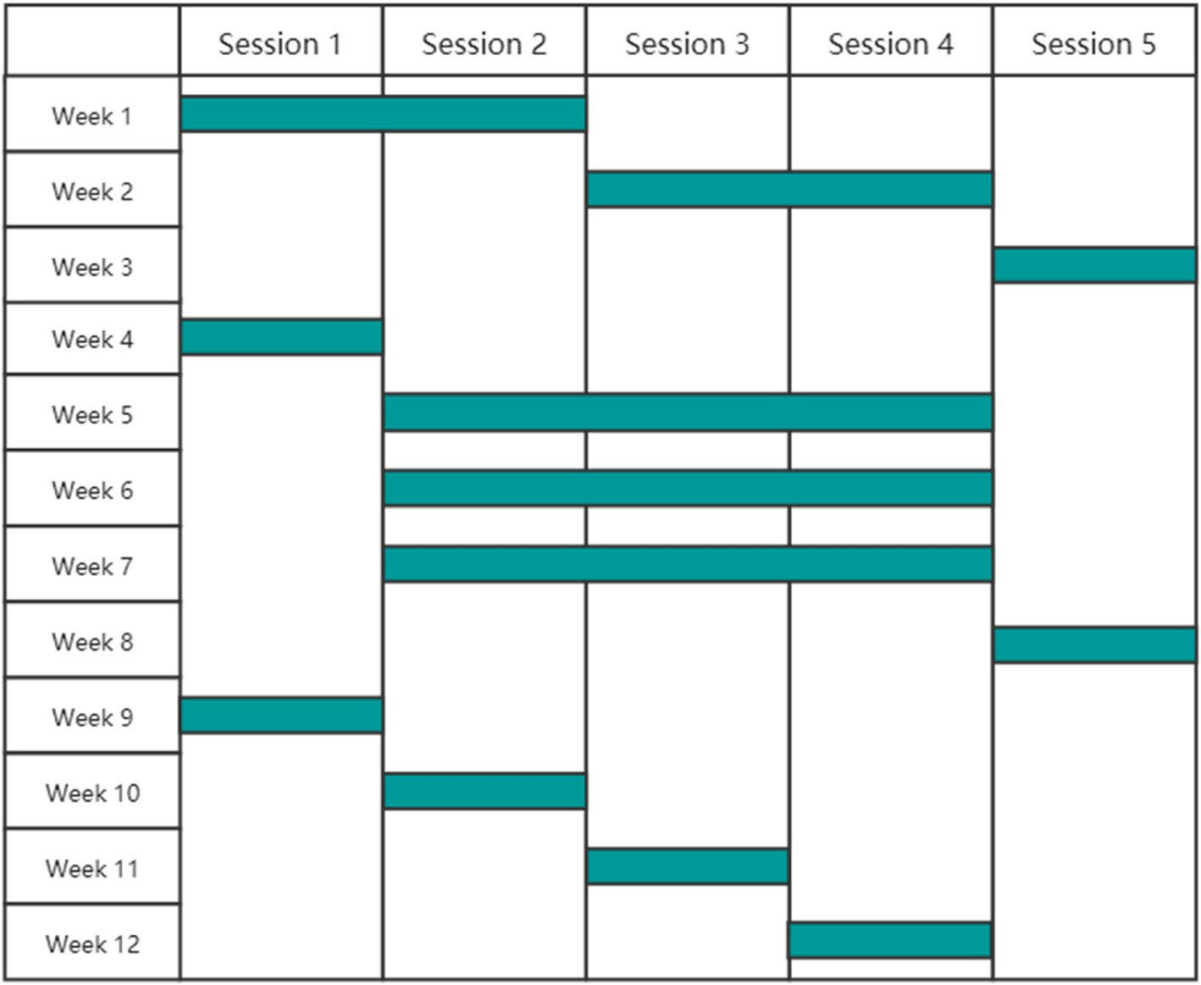
CBT intervention schedule

Interventions will be delivered by WeChat MiniApp: “CBT GDM” (developed by Hangzhou Kang Sheng Health Consulting CO., LTD). This includes 5 sessions. The contents are designed and reviewed by endocrinologists and psychiatrists from the First People’s Hospital of Kunshan affiliated with Jiangsu University, Kunshan, Jiangsu Province, China. Participants will study according to a learning schedule which is divided into a strong intervention period (1–3 weeks) and a regular intervention period (4–12 months) (see Fig. 1).

**Table:** 

***Session 1 objectives:***	To explain the CBT process To describe the correlation between CBT and glucose management To learn mindfulness mediation
***Session 2 objectives:***	To discuss clinical knowledge regarding GDM, including the onset, development and outcome of hyperglycemia To set the goal of therapy of hyperglycemia and learn how to monitor To describe standard pharmacological and non-pharmacological treatments To identify the importance of postpartum glucose management To understand the importance of compliance
***Session 3 objectives:***	To evaluate the significance of diet in lipid-lowering To recognize high-risk foods (such as foods high in sugar) To recognize healthy foods (such as foods high in fiber) To create a postpartum diet plan To demonstrate self-management of healthy diets
***Section 4 objectives:***	To describe the relationship between emotion and blood glucose during pregnancy To recognize how to accept negative emotions To recognize how to praise for positive emotions To implement emotions management
***Section 5 objectives:***	To analyze poor glucose control cases To reflect on positive and negative behavioral changes

### Criteria for discontinuing or modifying allocated interventions

In the following circumstances, dropping out of the treatment program will be considered. At this point, participants will not be regarded to have dropped out of the experiment, and they will be eligible for protocol assessments unless they withdraw their consent for assessments (as described below).

- 1) If the participant requests that the protocol treatment was discontinued.
- 2) If the participant is unable to continue the protocol treatment due to adverse events.
- 3) When the investigator believes that the risk of continuing the protocol treatment outweighs the benefits for any reason.
- 4) When the investigator believes that continuing the protocol treatment is not appropriate for any other reasons.

### Adherence

Participants in the intervention group will use the WeChat MiniApp to log in and complete the intervention sessions according to the schedule. The completion of each session will be tracked by “GDM ASCVD.” The “clock-in” function in the “GDM ASCVD” app will provide positive and constructive feedback to participants to enhance adherence. Participants will be able to “clock in” from the dashboard to track their exercise and eating habits on a regular basis.

### Ancillary for post-trial care

With regard to the nature of mobile-based patient education, participants are unlikely to be harmed. In the event that a risk of injury is discovered, the principal investigator will intervene to reduce the risk of harm. If any harm is discovered, the research team will compensate the participants appropriately if necessary.

### Outcomes

#### Primary outcome measures

The primary endpoint is the percentage of fasting blood glucose, which is defined as the percentage of blood glucose levels within a normal range [17]. Glucose monitoring data is collected twice weekly after enrollment [time frame: from randomization (day 0) to V5 (week 12) for a total of 3 months of treatment]. The selected examination days shall be at least 2 days apart. Each examination day should contain the following self-monitoring data.

**Table:** 

	Type	Measuring time	Normal range
1	Fasting blood glucose	Before breakfast	3.3–5.3 mmol/L
2	1-h postprandial blood glucose	After breakfast	3.3–7.8 mmol/L
3	2-h postprandial blood glucose	After breakfast	3.3–6.7 mmol/L
4	1-h postprandial blood glucose	After lunch	3.3–7.8 mmol/L
5	2-h postprandial blood glucose	After lunch	3.3–6.7 mmol/L
6	1-h postprandial blood glucose	After dinner	3.3–7.8 mmol/L
7	2-h postprandial blood glucose	After dinner	3.3–6.7 mmol/L

#### Secondary outcome measures

- 1) The percentage of blood glucose levels collected within the normal range on each selected examination day.
- 2) Loss of self-monitoring rate.
- 3) HbA1c.
- 4) Mean score of Diagnostic and Statistical Manual of Mental Disorders (DSM-5) assessment. DSM–5 is developed as a standard classification system for mental disorders. It provides a guideline of structured clinical interviews utilized to diagnose depression [18]
- 5) Mean score of General Self-Efficacy Scale (GSE). The GSE is a psychometric scale that measures the confidence in one’s capacity to deal with a wide range of life’s issues. The test consists of ten items that describe attitudes toward barriers and are assessed on a scale of 0 (least optimal) to 4 (very optimal). A version in Chinese translation has been approved [19]
- 6) Any insulin dose prescribed.
- 7) Case number of low-birth-weight neonates. Low birth weight is defined as the first weight recorded after the birth of less than 2500 g (up to and including 2499 g) [20]
- 8) Case number of neonatal macrosomia. Neonatal macrosomia is defined as the first weight recorded after the birth of more than 4000 g (more than and including 4000 g) [21].
- 9) Hypoglycemic events in neonates (blood glucose < 2.2 µmol/L).
- 10) Other fetal adverse events.
- 11) Case number of GDM comorbidity developed during the intervention period.
- 12) Case number of cesarean delivery.
- 13) Total expenditures for delivery.

### Participant timeline

**Fig. 2:**
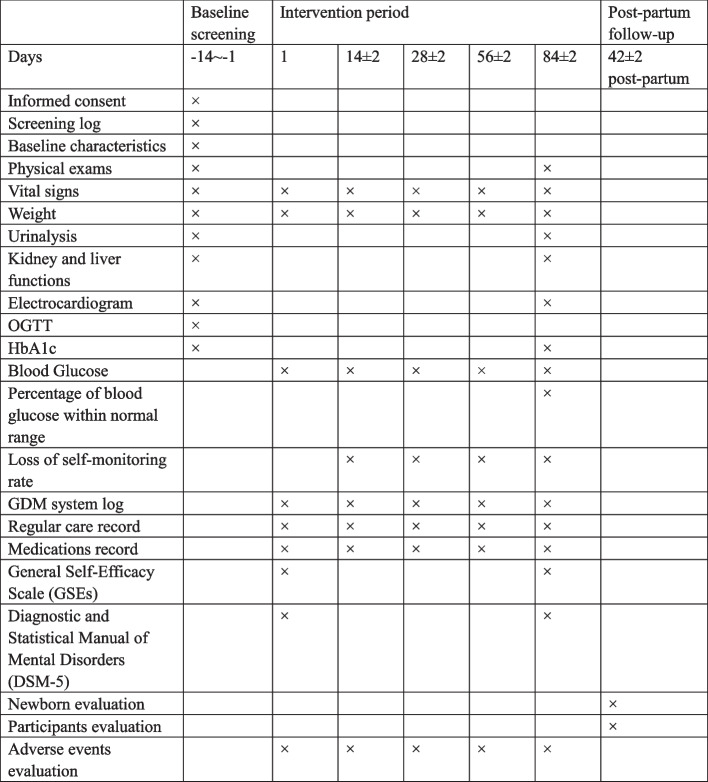
Participant timeline

The participant timeline is shown in Fig. 2.

### Sample size

Considering the primary outcome variable as the difference in the percentage of blood glucose within normal range between two groups, an absolute difference of 10% in the percentage of blood glucose within normal range is expected. Power was set at 0.8, the one-sided significance level at 0.05, and the confidence interval (CI) at 95%. Eighty subjects per group were calculated according to a 1:1 ratio of random grouping. A total sample size 100 was needed in each group that to account for an additional 20% in attrition size.

### Recruitment

Participant recruitment began in October 2021 and is expected to be completed in November 2021. We will recruit participants at the First People’s Hospital of Kunshan affiliated with Jiangsu University, Kunshan, Jiangsu Province, China. Once recruited, researchers will provide guidance on how to use the MiniApp.

### Assignment of interventions: allocation

#### Sequence generation

In this randomized controlled trial, participants will be randomized into either intervention or control arm with an allocation ratio of 1:1. The allocation will be generalized by the establishment of EDC Trial Data clinical trials centralized randomization system. Researchers will enter the system with account numbers and passwords. A randomized number generated by inputting patient information will be used to track each patient.

#### Concealment mechanism

Allocation concealment will be guaranteed through the following mechanism. Staff will log in to the EDC Trial Data cloud and enter the information for the participants. The EDC Trial Data system will randomly assign participants to either group in a 1:1 ratio. A patient profile will be generated after the necessary baseline information has been entered. Account number and password are required when assessing data.

#### Implementation

The allocation sequence will be generated by EDC centralized randomization system. YP will be in charge of enrolling the patients. The Trial Management Committee (TMC) will assign participants to interventions.

### Assignment of interventions: blinding

Participants and investigators will be aware of the intervention that has been assigned to them in this open-label trial. Because of the nature of the patient-reported outcome measures, not all outcome evaluations will be blinded. During the analysis, the statistician will be blinded to the allocation. The procedure for unblinding is not applicable as this is an open-label trial.

### Data collection and management

#### Data collection

Data will be collected utilizing EDC at study site. Data collection takes place at seven points: baseline, day 1, day 14 (± 2), day 28 (± 2), day 56 (± 2), day 84 (± 2) during intervention period, and day 42 (± 2) post-partum. Collected data will be stored in the form of an electronic case report form. The clinical research coordinator (CRC) will complete the electronic case report form. The investigators will be informed of any changes made by CRC and system/edit check automatically done by the system. Authorized investigators will be permitted to access the study computers and evaluate the data throughout the study. If any missing data is detected via the EDC system, the researcher will contact the participants on the telephone.

#### Data management

Study teams have a responsibility to self-monitor study processes and data. This self-monitoring can ensure a well-run trial and to identify and mitigate issues before they are identified by monitoring entities, which can result in time-consuming fixes. Internal monitoring includes monitoring for proper informed consent documentation/records, eligibility criteria, and data quality. Monitoring is usually conducted by interested parties involved in the research to identify issues and to improve processes.

A unique identification code will be generated when analyzing data to ensure security. Errors or missing data information will be sent to data managers (DM) in data query reports. DM receiving the inquiry will check the original records to determine the correction. Original paperwork and signed informed consent forms will be stored in locked file cabinets.

#### Confidentiality

Personal information will be collected, shared, and maintained following ICH GCP.

### Statistical methods

#### Statistical methods for primary and secondary outcomes

Categorical variables will be expressed in numerical values with their respective percentages and compared using the chi-squared test at baseline. Continuous variables will be expressed as mean ± SD and compared using the *t*-test. The significance of primary and secondary endpoints will be evaluated with the paired-samples *t*-test. A *p*-value < 0.05 indicates statistical significance. All data will be analyzed with the SPSS.

All patients who have received CBT for at least 4 weeks will be included in the full analysis set (FAS). Depending on the time investigated, the per-protocol (PP) analysis set will be made up of patients who complete the 6-month follow-up examinations. The FAS will be used as the basis for the primary analysis. If there is a discrepancy of greater than 10% between the FAS and PP samples, the analysis will be repeated for the PP analysis set. The safety population will include patients in the FAS. Unless otherwise noted, all analysis will be done in both the FAS and PP analysis sets. No additional analysis will be conducted.

There will be no replacement or imputation of missing values. All analysis will be based solely on observed cases. Patients who loss to follow-up will be considered to have dropped out of therapy. Only the PP patients will be used in a sensitivity analysis to test the impact of this assumption.

### Oversight and monitoring

#### Composition of the coordinating center and trial steering committee

Design and conduct of the study: preparation of protocol and amendments; preparation of IB (investigators brochure) and CRFs (case report forms); reports on studies to be published; composition of TMC’s members.

SC (steering committee): final protocol agreement; patient recruitment and communication with the lead investigator; reviewing the trial’s progress and, if necessary, consenting to adjustments to the protocol and/or investigators brochure to help the study run smoothly. The steering group will include all principal investigators. A regional coordinator will be chosen from among the principal investigators at each site.

Trial Management Committee (TMC) (principal investigator, research physician, administrator): study planning; organization of steering committee meetings; report of adverse events to the Chinese National Adverse Reaction Monitoring Center; responsible for the trial master file; budget management and contractual difficulties with individual centers, randomization; data verification. TMC will examine the 12 monthly feedback forms and schedule site visits; data manager: data entry and maintenance of the trial’s IT system; data verification.

Lead investigators: recruitment; data collection; CRF completion, as well as study patient follow-up and adherence to the study protocol and IB. A lead investigator will be assigned to each participating center and will be responsible for patient identification.

#### Adverse event reporting and harms

The type and frequencies of any adverse event will be reported to the Chinese National Adverse Reaction Monitoring Center.

#### Frequency and plans for auditing trial conduct

Audit procedures will comply with the ICH GCP (Guideline for Good Clinical Practice of the International Conference on Harmonisation) and regulatory requirements. The biannual audit was conducted by the ethics committee of the hospital, and the audit process was independent of the sponsor. The steering committee will determine any revisions of the protocol. The amended protocol will also be submitted to the Medical Ethics Committee of the Sir Run Run Shaw Hospital of Zhejiang University and reported to the participants as necessary.

The findings of the study will be presented to the public at academic conferences. The steering group will decide on authorship. The order of the authors will be determined by the contributions of each member.

## Discussion

This article presents a detailed description of a parallel, randomized control trial, designed to evaluate the effectiveness of CBT to reduce hyperglycemia in GDM patients. Lifestyle intervention is recommended as the first-line treatment for GDM per guidelines. To accomplish the primary goal of controlling blood glucose, we consider the mobile-based CBT intervention as an available and accessible method with regard to how to promote healthy habits, and as a result, this will potentially lower blood glucose in ASCVD patients. We believe that mobile-based CBT lifestyle intervention will be potentially effective in lowering blood glucose levels in ASCVD patients.

This study has some critical implications. The novel mobile-based lifestyle intervention is expected to reduce glucose level at a population level due to the high accessibility and availability of technologies. Moreover, this study can potentially demonstrate CBT-based lifestyle intervention’s efficacy in infant outcomes and be utilized as a future guidance for lifestyle intervention in GDM patients.

There are some limitations of this study. First, this study excludes pregnant women whose past medical history include diabetes and only investigates women developing diabetes after diabetes. Future studies could involve a larger patient group. Second, it is impossible to blind participants and staff (excluding statistician) in psychotherapy studies due to the nature of the research. As a result, therapy expectancy effects may be a source of bias.

## Trial status

Protocol version: original; version 1.1 20,210,920.

Recruitment start date: October 1, 2021.

Anticipated completion of recruitment: November 1, 2021.

## Data Availability

The data generated by the study will eventually belong to the study team. If the subjects are infringed due to data disclosure in the trial, the research team will provide them with appropriate compensation if necessary.

## Abbreviations

CBT: Cognitive behavioral therapy
CRC: Clinical research coordinator
CRFs: Case report forms
DM: Data manager
DRD: Diabetes-related distress
DSM-5: Diagnostic and Statistical Manual of Mental Disorders
FAS: Full analysis set
GDM: Gestational diabetes mellitus
GSEs: GENERAL Self-Efficacy Scale (GSEs)
HbA1c: Hemoglobin A1c
IB: Investigators brochure
ICH GCP: Guideline for Good Clinical Practice of the International Conference on Harmonisation
MeSH: Medical Subject Headings
SC: Steering committee
SPIRIT: Standard Protocol ItemsRecommendations for Interventional Trials
T2DM: Type 2 diabetes mellitus
TMC: Trial management committee

## References

[1] Dabelea D, Hanson RL, Lindsay RS, Pettitt DJ, Imperatore G, Gabir MM, Roumain J, Bennett PH, Knowler WC (2000). Intrauterine exposure to diabetes conveys risks for type 2 diabetes and obesity: a study of discordant sibships. Diabetes.

[2] Holmes VA, Young IS, Patterson CC, Pearson DWM, Walker JD, Maresh MJA, McCance DR (2011). Optimal glycemic control, pre-eclampsia, and gestational hypertension in women with type 1 diabetes in the diabetes and pre-eclampsia intervention trial. Diabetes Care.

[3] Management of Diabetes in Pregnancy (2020). Standards of Medical Care in Diabetes—2021. Diabetes Care.

[4] Diabetes Society of Chinese Medical Association (2021). Guideline for the prevention and treatment of type 2 diabetes mellitus in China (2020 edition) (in Chinese). Chinese J Diabetes.

[5] Hod M, Kapur A, Sacks DA, Hadar E, Agarwal M, Di Renzo GC, Roura LC, McIntyre HD, Morris JL, Divakar H (2015). The International Federation of Gynecology and Obstetrics (FIGO) Initiative on Gestational Diabetes Mellitus: A Pragmatic Guide for Diagnosis, Management, and Care#. Int J Gynecol Obstet.

[6] Oskovi-Kaplan ZA, Ozgu-Erdinc AS. Management of Gestational Diabetes Mellitus. In: Islam, M.S. (eds) Diabetes: from Research to Clinical Practice. Advances in Experimental Medicine and Biology. Cham: Springer; 2020. p. 257–72. 10.1007/5584_2020_552 32548833

[7] Beck AT (2016). Cognitive Therapy: Nature and relation to behavior therapy – republished article. Behav Ther.

[8] Friedman HS (2014). Oxford Handbook of Health Psychology.

[9] Chen DA, Wang PC, Lin YH (2019). The relationship among perceived stress, self-efficacy, self-care behaviors, psychological distress and type D personality in type 2 diabetes mellitus. J Intern Med Taiwan.

[10] Lakerveld J, Bot SD, Chinapaw MJ, van Tulder MW, van Oppen P, Dekker JM, Nijpels G (2008). Primary prevention of diabetes mellitus type 2 and cardiovascular diseases using a cognitive behavior program aimed at lifestyle changes in people at risk: design of a randomized controlled trial. BMC Endocrine Disorders.

[11] ACOG Practice Bulletin No (2001). 30: Gestational Diabetes. Obstet Gynecol.

[12] Tunsuchart K, Lerttrakarnnon P, Srithanaviboonchai K, Likhitsathian S, Skulphan S (2020). Benefits of brief group cognitive behavioral therapy in reducing diabetes-related distress and HbA1c in uncontrolled type 2 diabetes mellitus patients in Thailand. Int J Environ Res Public Health.

[13] Weinger K (2011). The effect of a structured behavioral intervention on poorly controlled diabetes. Arch Intern Med.

[14] Lakerveld J, Bot SD, Chinapaw MJ, van Tulder MW, Kostense PJ, Dekker JM, Nijpels G (2013). Motivational interviewing and problem solving treatment to reduce type 2 diabetes and cardiovascular disease risk in real life: a randomized controlled trial. Int J Behav Nutr Phys Act.

[15] Li Y, Long D, Liu J, Qiu D, Wang J, Cheng X, Yang X, Li R, Wang G (2020). Gestational diabetes mellitus in women increased the risk of neonatal infection via inflammation and autophagy in the placenta. Medicine.

[16] Chan AW, Tetzlaff JM, Gotzsche PC, Altman DG, Mann H, Berlin JA, Dickersin K, Hrobjartsson A, Schulz KF, Parulekar WR, Krleza-Jeric K, Laupacis A, Moher D (2013). SPIRIT 2013 explanation and elaboration: guidance for protocols of clinical trials. BMJ.

[17] Time-In-Range. https://diatribe.org/time-range. Accessed 8 Apr 2022.

[18] American Psychiatric Association. Diagnostic and Statistical Manual of Mental Disorders. 5th Ed. Washington: American Psychiatric Association; 2013.

[19] Cheung S-K, Sun SY K (1999). Assessment of optimistic self-beliefs: further validation of the Chinese Version of the General Self-Efficacy Scale. Psychological Reports.

[20] Rust J (2010). Updating the International Classification of Diseases and Related Health Problems, Tenth Revision (ICD-10). Health Information Management Journal.

[21] Martin JA, Hamilton BE, Sutton PD, Ventura SJ, Menacker F, Kirmeyer S (2006). Births: final data for 2004. Natl Vital Stat Rep.

